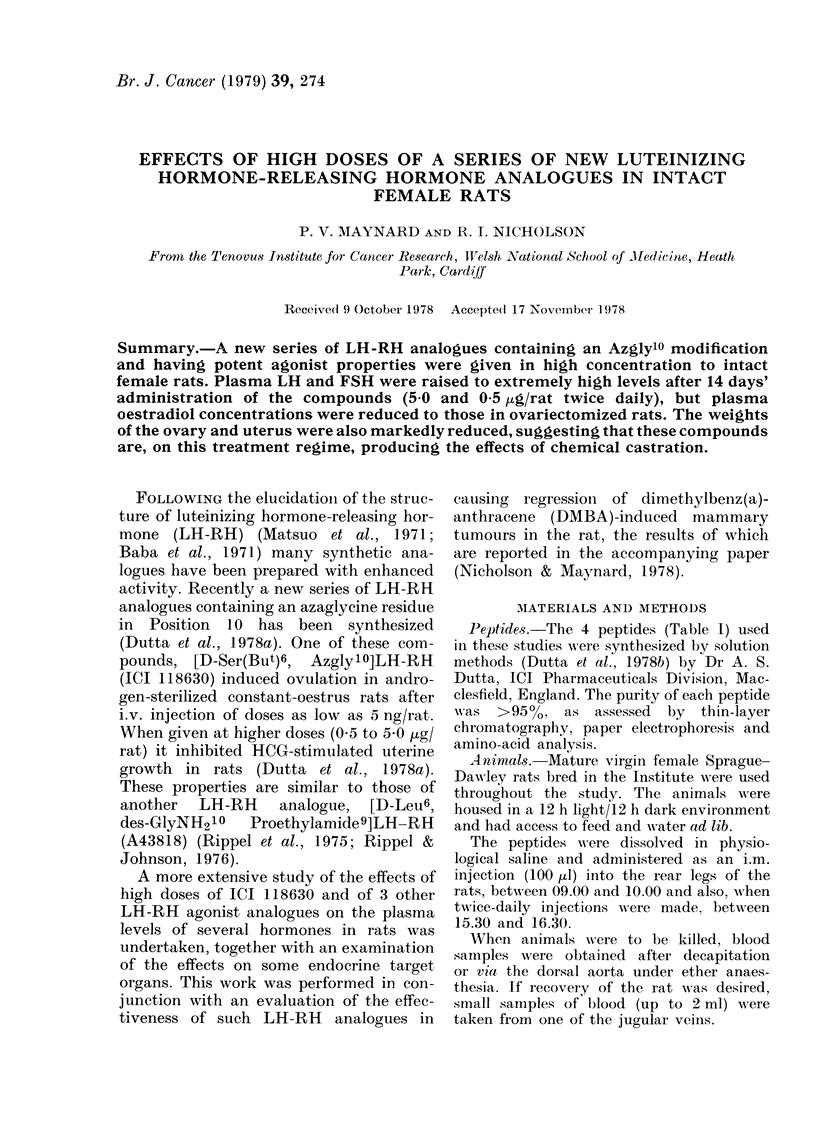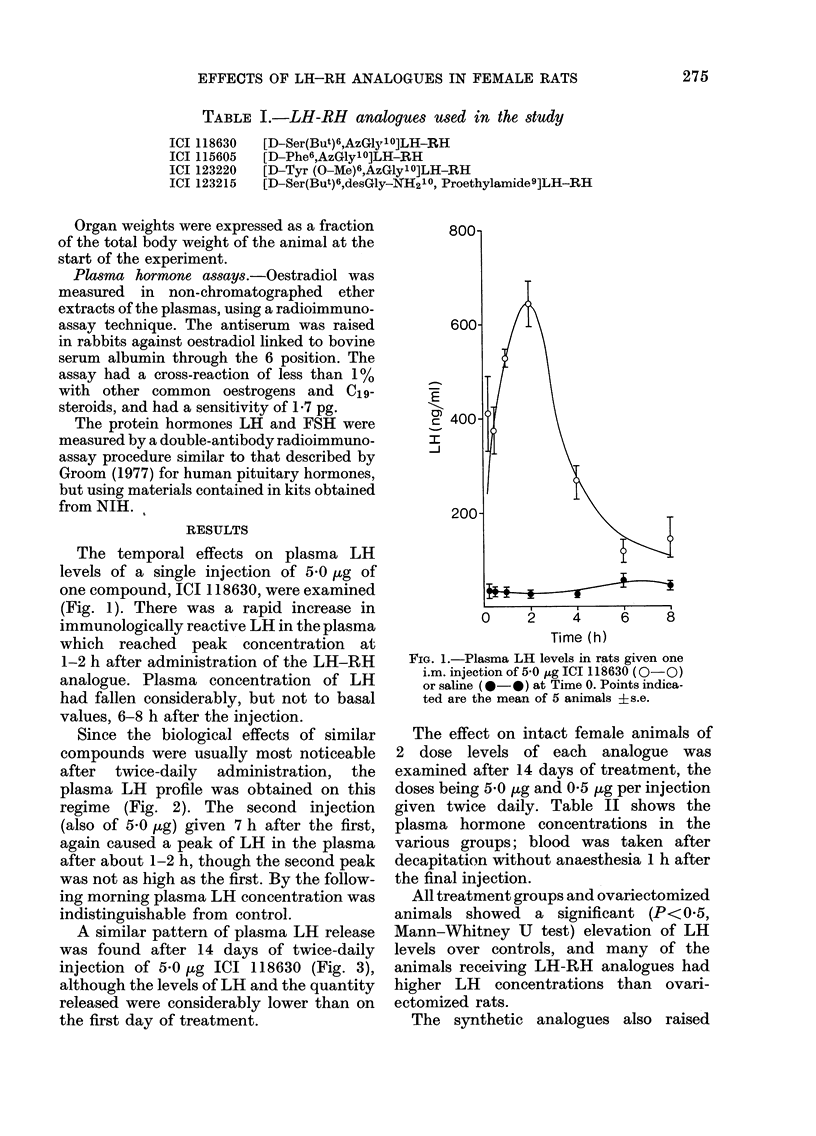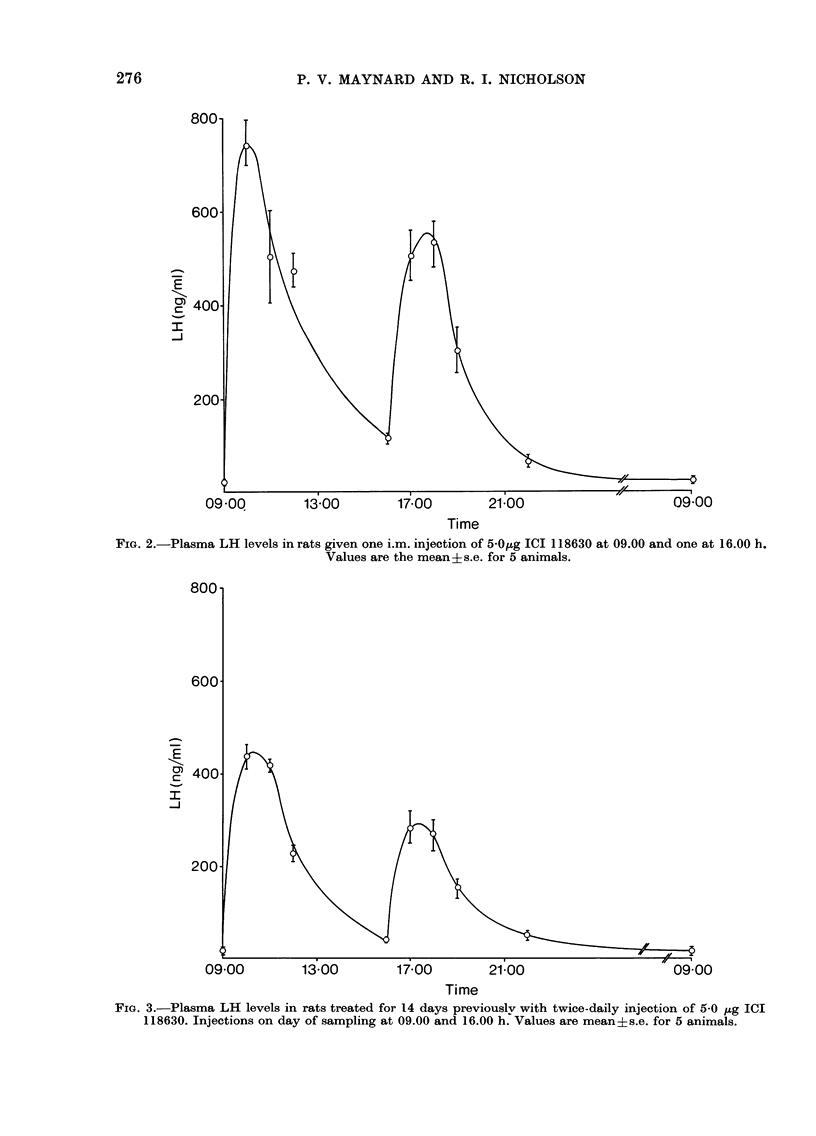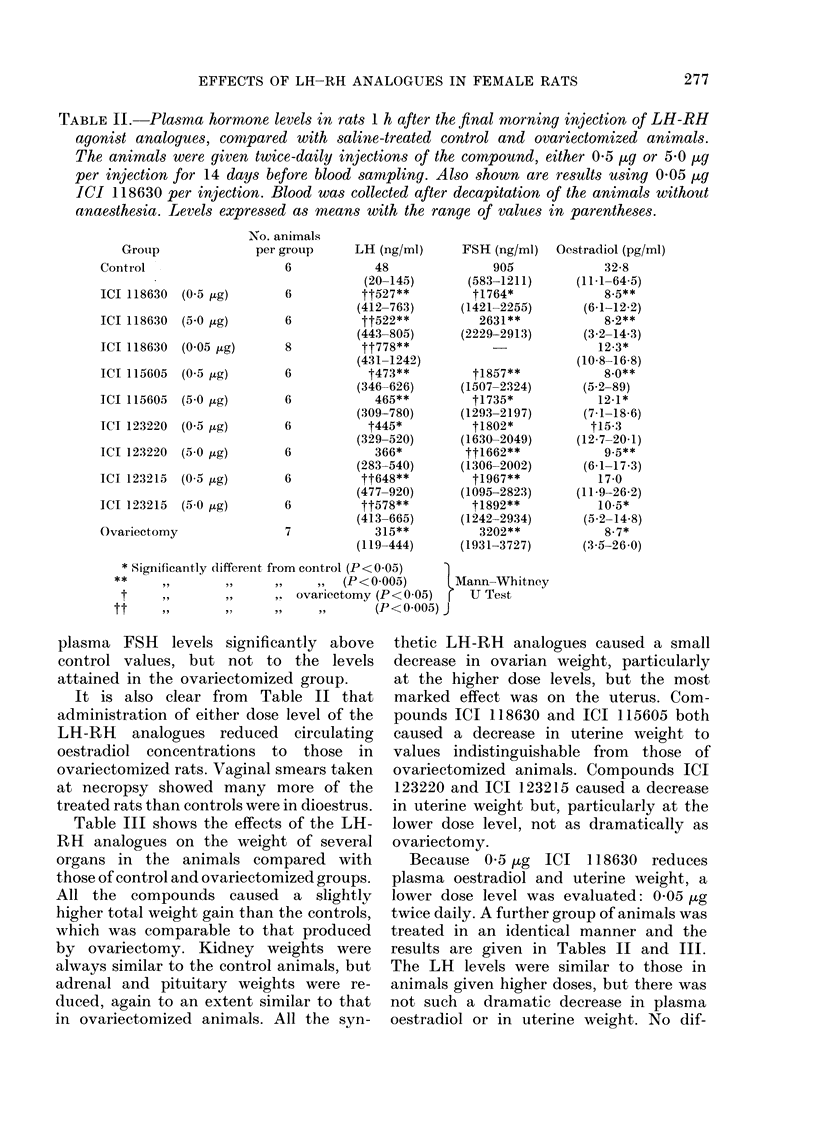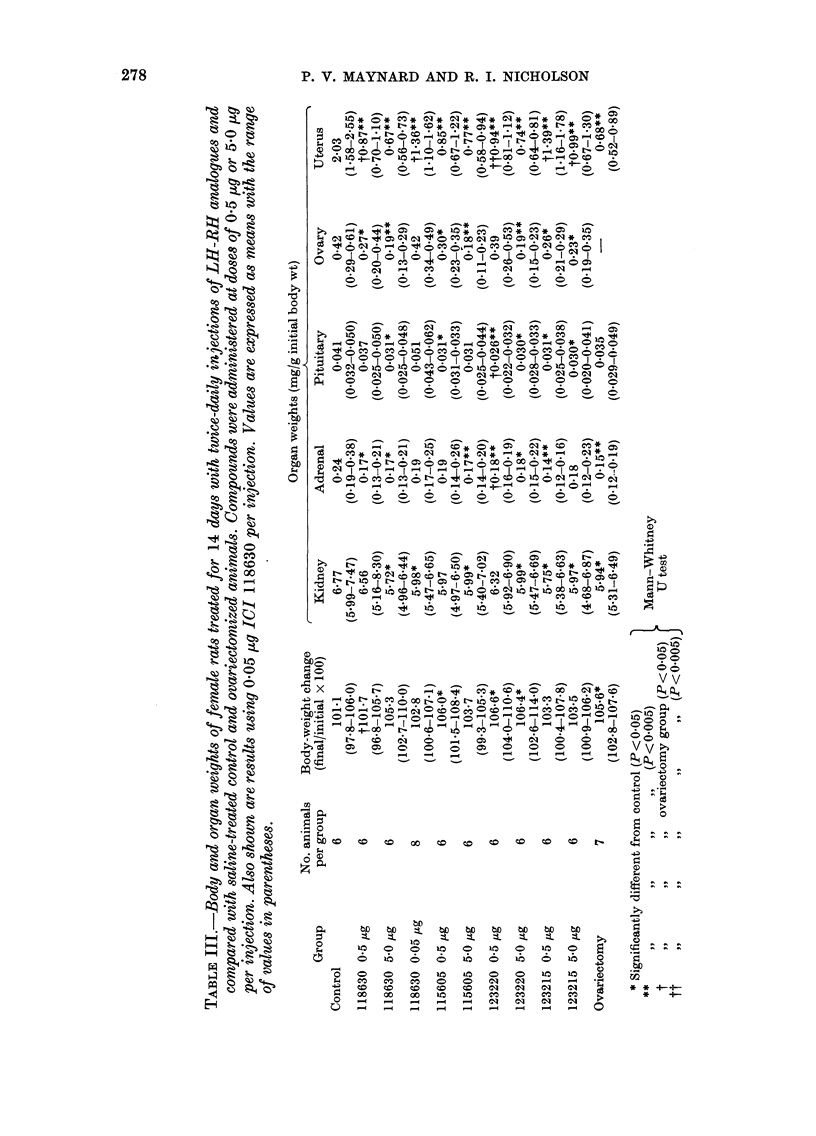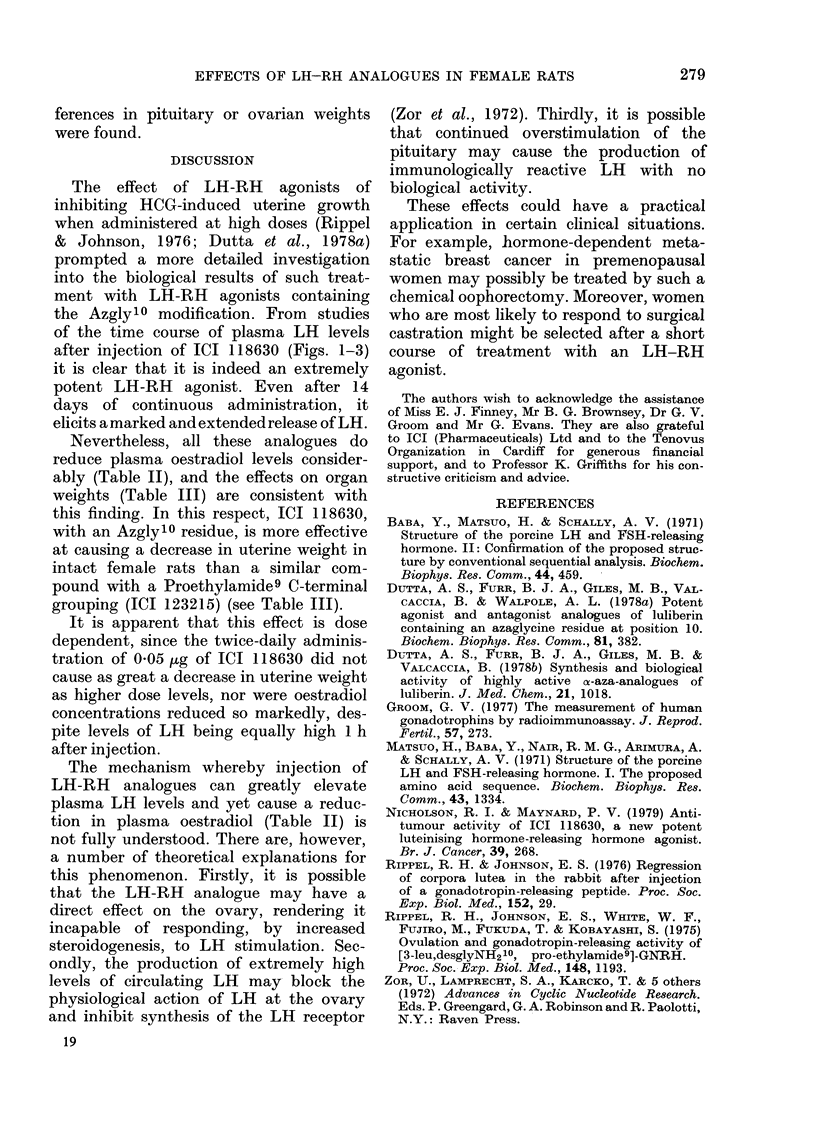# Effects of high doses of a series of new luteinizing hormone-releasing hormone analogues in intact female rats.

**DOI:** 10.1038/bjc.1979.51

**Published:** 1979-03

**Authors:** P. V. Maynard, R. I. Nicholson

## Abstract

A new series of LH-RH analogues containing an Azgly10 modification and having potent agonist properties were given in high concentration to intact female rats. Plasma LH and FSH were raised to extremely high levels after 14 days' administration of the compounds (5.0 and 0.5 microgram/rat twice daily), but plasma oestradiol concentrations were reduced to those in ovariectomized rats. The weights of the ovary and uterus were also markedly reduced, suggesting that these compounds are, on this treatment regime, producing the effects of chemical castration.


*Br. J. Cancer* (1979) **39**, 274

### EFFECTS OF HIGH DOSES OF A SERIES OF NEW LUTEINIZING HORMONE-RELEASING HORMONE ANALOGUES IN INTACT FEMALE RATS

P. V. MAYNARD AND R. I. NICHOLSON

*From the Tenovus Institute for Cancer Research, Welsh National School of Medicine, Heath Park, Cardiff*

Received 9 October 1978 Accepted 17 November 1978

**Summary.—A new series of LH-RH analogues containing an Azgly$^{10}$ modification and having potent agonist properties were given in high concentration to intact female rats. Plasma LH and FSH were raised to extremely high levels after 14 days' administration of the compounds (5·0 and 0·5 $\mu$g/rat twice daily), but plasma oestradiol concentrations were reduced to those in ovariectomized rats. The weights of the ovary and uterus were also markedly reduced, suggesting that these compounds are, on this treatment regime, producing the effects of chemical castration.**

FOLLOWING the elucidation of the structure of luteinizing hormone-releasing hormone (LH-RH) (Matsuo *et al.*, 1971; Baba *et al.*, 1971) many synthetic analogues have been prepared with enhanced activity. Recently a new series of LH-RH analogues containing an azaglycine residue in Position 10 has been synthesized (Dutta *et al.*, 1978*a*). One of these compounds, [D-Ser(Bu$^t$)$^6$, Azgly$^{10}$]LH-RH (ICI 118630) induced ovulation in androgen-sterilized constant-oestrus rats after i.v. injection of doses as low as 5 ng/rat. When given at higher doses (0·5 to 5·0 $\mu$g/rat) it inhibited HCG-stimulated uterine growth in rats (Dutta *et al.*, 1978*a*). These properties are similar to those of another LH-RH analogue, [D-Leu$^6$, des-Gly$NH_2$$^{10}$ Proethylamide$^9$]LH-RH (A43818) (Rippel *et al.*, 1975; Rippel & Johnson, 1976).

A more extensive study of the effects of high doses of ICI 118630 and of 3 other LH-RH agonist analogues on the plasma levels of several hormones in rats was undertaken, together with an examination of the effects on some endocrine target organs. This work was performed in conjunction with an evaluation of the effectiveness of such LH-RH analogues in causing regression of dimethylbenz(a)-anthracene (DMBA)-induced mammary tumours in the rat, the results of which are reported in the accompanying paper (Nicholson & Maynard, 1978).

### MATERIALS AND METHODS

*Peptides.*—The 4 peptides (Table I) used in these studies were synthesized by solution methods (Dutta *et al.*, 1978*b*) by Dr A. S. Dutta, ICI Pharmaceuticals Division, Macclesfield, England. The purity of each peptide was >95%, as assessed by thin-layer chromatography, paper electrophoresis and amino-acid analysis.

*Animals.*—Mature virgin female Sprague–Dawley rats bred in the Institute were used throughout the study. The animals were housed in a 12 h light/12 h dark environment and had access to feed and water *ad lib.*

The peptides were dissolved in physiological saline and administered as an i.m. injection (100 $\mu$l) into the rear legs of the rats, between 09.00 and 10.00 and also, when twice-daily injections were made, between 15.30 and 16.30.

When animals were to be killed, blood samples were obtained after decapitation or *via* the dorsal aorta under ether anaesthesia. If recovery of the rat was desired, small samples of blood (up to 2 ml) were taken from one of the jugular veins.

TABLE I.—*LH-RH analogues used in the study*

| | |
|---|---|
| ICI 118630 | [D–Ser(But)$^6$,AzGly$^{10}$]LH–RH |
| ICI 115605 | [D–Phe$^6$,AzGly$^{10}$]LH–RH |
| ICI 123220 | [D–Tyr (O–Me)$^6$,AzGly$^{10}$]LH–RH |
| ICI 123215 | [D–Ser(But)$^6$,desGly–$NH_2{}^{10}$, Proethylamide$^9$]LH–RH |

Organ weights were expressed as a fraction of the total body weight of the animal at the start of the experiment.

*Plasma hormone assays.*—Oestradiol was measured in non-chromatographed ether extracts of the plasmas, using a radioimmunoassay technique. The antiserum was raised in rabbits against oestradiol linked to bovine serum albumin through the 6 position. The assay had a cross-reaction of less than 1% with other common oestrogens and $C_{19}$-steroids, and had a sensitivity of 1·7 pg.

The protein hormones LH and FSH were measured by a double-antibody radioimmunoassay procedure similar to that described by Groom (1977) for human pituitary hormones, but using materials contained in kits obtained from NIH.

RESULTS

The temporal effects on plasma LH levels of a single injection of 5·0 μg of one compound, ICI 118630, were examined (Fig. 1). There was a rapid increase in immunologically reactive LH in the plasma which reached peak concentration at 1–2 h after administration of the LH–RH analogue. Plasma concentration of LH had fallen considerably, but not to basal values, 6–8 h after the injection.

Since the biological effects of similar compounds were usually most noticeable after twice-daily administration, the plasma LH profile was obtained on this regime (Fig. 2). The second injection (also of 5·0 μg) given 7 h after the first, again caused a peak of LH in the plasma after about 1–2 h, though the second peak was not as high as the first. By the following morning plasma LH concentration was indistinguishable from control.

A similar pattern of plasma LH release was found after 14 days of twice-daily injection of 5·0 μg ICI 118630 (Fig. 3), although the levels of LH and the quantity released were considerably lower than on the first day of treatment.

FIG. 1.—Plasma LH levels in rats given one i.m. injection of 5·0 μg ICI 118630 (○—○) or saline (●—●) at Time 0. Points indicated are the mean of 5 animals ±s.e.

The effect on intact female animals of 2 dose levels of each analogue was examined after 14 days of treatment, the doses being 5·0 μg and 0·5 μg per injection given twice daily. Table II shows the plasma hormone concentrations in the various groups; blood was taken after decapitation without anaesthesia 1 h after the final injection.

All treatment groups and ovariectomized animals showed a significant ($P<0{\cdot}5$, Mann–Whitney U test) elevation of LH levels over controls, and many of the animals receiving LH-RH analogues had higher LH concentrations than ovariectomized rats.

The synthetic analogues also raised

FIG. 2.—Plasma LH levels in rats given one i.m. injection of 5·0μg ICI 118630 at 09.00 and one at 16.00 h. Values are the mean ± s.e. for 5 animals.

FIG. 3.—Plasma LH levels in rats treated for 14 days previously with twice-daily injection of 5·0 μg ICI 118630. Injections on day of sampling at 09.00 and 16.00 h. Values are mean ± s.e. for 5 animals.

TABLE II.—*Plasma hormone levels in rats* 1 *h after the final morning injection of LH-RH agonist analogues, compared with saline-treated control and ovariectomized animals. The animals were given twice-daily injections of the compound, either* 0·5 *μg or* 5·0 *μg per injection for* 14 *days before blood sampling. Also shown are results using* 0·05 *μg ICI* 118630 *per injection. Blood was collected after decapitation of the animals without anaesthesia. Levels expressed as means with the range of values in parentheses.*

| Group | | No. animals per group | LH (ng/ml) | FSH (ng/ml) | Oestradiol (pg/ml) |
|---|---|---|---|---|---|
| Control | | 6 | 48 (20–145) | 905 (583–1211) | 32·8 (11·1–64·5) |
| ICI 118630 | (0·5 μg) | 6 | ††527** (412–763) | †1764* (1421–2255) | 8·5** (6·1–12·2) |
| ICI 118630 | (5·0 μg) | 6 | ††522** (443–805) | 2631** (2229–2913) | 8·2** (3·2–14·3) |
| ICI 118630 | (0·05 μg) | 8 | ††778** (431–1242) | — | 12·3* (10·8–16·8) |
| ICI 115605 | (0·5 μg) | 6 | †473** (346–626) | †1857** (1507–2324) | 8·0** (5·2–89) |
| ICI 115605 | (5·0 μg) | 6 | 465** (309–780) | †1735* (1293–2197) | 12·1* (7·1–18·6) |
| ICI 123220 | (0·5 μg) | 6 | †445* (329–520) | †1802* (1630–2049) | †15·3 (12·7–20·1) |
| ICI 123220 | (5·0 μg) | 6 | 366* (283–540) | ††1662** (1306–2002) | 9·5** (6·1–17·3) |
| ICI 123215 | (0·5 μg) | 6 | ††648** (477–920) | †1967** (1095–2823) | 17·0 (11·9–26·2) |
| ICI 123215 | (5·0 μg) | 6 | ††578** (413–665) | †1892** (1242–2934) | 10·5* (5·2–14·8) |
| Ovariectomy | | 7 | 315** (119–444) | 3202** (1931–3727) | 8·7* (3·5–26·0) |

* Significantly different from control ($P<0{\cdot}05$)

** „ „ „ „ ($P<0{\cdot}005$)

† „ „ „ ovariectomy ($P<0{\cdot}05$)

†† „ „ „ „ ($P<0{\cdot}005$)

Mann–Whitney U Test plasma FSH levels significantly above control values, but not to the levels attained in the ovariectomized group.

It is also clear from Table II that administration of either dose level of the LH-RH analogues reduced circulating oestradiol concentrations to those in ovariectomized rats. Vaginal smears taken at necropsy showed many more of the treated rats than controls were in dioestrus.

Table III shows the effects of the LH-RH analogues on the weight of several organs in the animals compared with those of control and ovariectomized groups. All the compounds caused a slightly higher total weight gain than the controls, which was comparable to that produced by ovariectomy. Kidney weights were always similar to the control animals, but adrenal and pituitary weights were reduced, again to an extent similar to that in ovariectomized animals. All the synthetic LH-RH analogues caused a small decrease in ovarian weight, particularly at the higher dose levels, but the most marked effect was on the uterus. Compounds ICI 118630 and ICI 115605 both caused a decrease in uterine weight to values indistinguishable from those of ovariectomized animals. Compounds ICI 123220 and ICI 123215 caused a decrease in uterine weight but, particularly at the lower dose level, not as dramatically as ovariectomy.

Because 0·5 μg ICI 118630 reduces plasma oestradiol and uterine weight, a lower dose level was evaluated: 0·05 μg twice daily. A further group of animals was treated in an identical manner and the results are given in Tables II and III. The LH levels were similar to those in animals given higher doses, but there was not such a dramatic decrease in plasma oestradiol or in uterine weight. No dif-

** " " " " ($P<0·005$)

†† " " " " " ($P<0·005$)

} Mann–Whitney U test ferences in pituitary or ovarian weights were found.

### DISCUSSION

The effect of LH-RH agonists of inhibiting HCG-induced uterine growth when administered at high doses (Rippel & Johnson, 1976; Dutta *et al.*, 1978*a*) prompted a more detailed investigation into the biological results of such treatment with LH-RH agonists containing the $Azgly^{10}$ modification. From studies of the time course of plasma LH levels after injection of ICI 118630 (Figs. 1–3) it is clear that it is indeed an extremely potent LH-RH agonist. Even after 14 days of continuous administration, it elicits a marked and extended release of LH.

Nevertheless, all these analogues do reduce plasma oestradiol levels considerably (Table II), and the effects on organ weights (Table III) are consistent with this finding. In this respect, ICI 118630, with an $Azgly^{10}$ residue, is more effective at causing a decrease in uterine weight in intact female rats than a similar compound with a $Proethylamide^{9}$ C-terminal grouping (ICI 123215) (see Table III).

It is apparent that this effect is dose dependent, since the twice-daily administration of 0·05 μg of ICI 118630 did not cause as great a decrease in uterine weight as higher dose levels, nor were oestradiol concentrations reduced so markedly, despite levels of LH being equally high 1 h after injection.

The mechanism whereby injection of LH-RH analogues can greatly elevate plasma LH levels and yet cause a reduction in plasma oestradiol (Table II) is not fully understood. There are, however, a number of theoretical explanations for this phenomenon. Firstly, it is possible that the LH-RH analogue may have a direct effect on the ovary, rendering it incapable of responding, by increased steroidogenesis, to LH stimulation. Secondly, the production of extremely high levels of circulating LH may block the physiological action of LH at the ovary and inhibit synthesis of the LH receptor (Zor *et al.*, 1972). Thirdly, it is possible that continued overstimulation of the pituitary may cause the production of immunologically reactive LH with no biological activity.

These effects could have a practical application in certain clinical situations. For example, hormone-dependent metastatic breast cancer in premenopausal women may possibly be treated by such a chemical oophorectomy. Moreover, women who are most likely to respond to surgical castration might be selected after a short course of treatment with an LH–RH agonist.

The authors wish to acknowledge the assistance of Miss E. J. Finney, Mr B. G. Brownsey, Dr G. V. Groom and Mr G. Evans. They are also grateful to ICI (Pharmaceuticals) Ltd and to the Tenovus Organization in Cardiff for generous financial support, and to Professor K. Griffiths for his constructive criticism and advice.

### REFERENCES

Baba, Y., Matsuo, H. & Schally, A. V. (1971) Structure of the porcine LH and FSH-releasing hormone. II: Confirmation of the proposed structure by conventional sequential analysis. *Biochem. Biophys. Res. Comm.*, **44**, 459.

Dutta, A. S., Furr, B. J. A., Giles, M. B., Valcaccia, B. & Walpole, A. L. (1978*a*) Potent agonist and antagonist analogues of luliberin containing an azaglycine residue at position 10. *Biochem. Biophys. Res. Comm.*, **81**, 382.

Dutta, A. S., Furr, B. J. A., Giles, M. B. & Valcaccia, B. (1978*b*) Synthesis and biological activity of highly active α-aza-analogues of luliberin. *J. Med. Chem.*, **21**, 1018.

Groom, G. V. (1977) The measurement of human gonadotrophins by radioimmunoassay. *J. Reprod. Fertil.*, **57**, 273.

Matsuo, H., Baba, Y., Nair, R. M. G., Arimura, A. & Schally, A. V. (1971) Structure of the porcine LH and FSH-releasing hormone. I. The proposed amino acid sequence. *Biochem. Biophys. Res. Comm.*, **43**, 1334.

Nicholson, R. I. & Maynard, P. V. (1979) Antitumour activity of ICI 118630, a new potent luteinising hormone-releasing hormone agonist. *Br. J. Cancer*, **39**, 268.

Rippel, R. H. & Johnson, E. S. (1976) Regression of corpora lutea in the rabbit after injection of a gonadotropin-releasing peptide. *Proc. Soc. Exp. Biol. Med.*, **152**, 29.

Rippel, R. H., Johnson, E. S., White, W. F., Fujiro, M., Fukuda, T. & Kobayashi, S. (1975) Ovulation and gonadotropin-releasing activity of [3-leu,desgly$NH_2^{10}$, pro-ethylamide$^{9}$]-GNRH. *Proc. Soc. Exp. Biol. Med.*, **148**, 1193.

Zor, U., Lamprecht, S. A., Karcko, T. & 5 others (1972) *Advances in Cyclic Nucleotide Research.* Eds. P. Greengard, G. A. Robinson and R. Paolotti, N.Y.: Raven Press.